# Association Between Maternal Weight Gain in Different Periods of Pregnancy and the Risk of Venous Thromboembolism: A Retrospective Case–Control Study

**DOI:** 10.3389/fendo.2022.858868

**Published:** 2022-07-18

**Authors:** Yuelin Wu, Jindan Pei, Lingling Dong, Zheying Zhou, Tianfan Zhou, Xiaobo Zhao, Ronghua Che, Zhimin Han, Xiaolin Hua

**Affiliations:** ^1^ Shanghai Key Laboratory of Maternal Fetal Medicine, Shanghai First Maternity and Infant Hospital, School of Medicine, Tongji University, Shanghai, China; ^2^ Obstetrics Department, Shanghai First Maternity and Infant Hospital, School of Medicine, Tongji University, Shanghai, China

**Keywords:** venous thromboembolic disease, pulmonary embolus, gestational weight gain, pregnancy outcomes, z-scores

## Abstract

**Background:**

Venous thromboembolism (VTE) remains an important cause of maternal deaths. Little is known about the associations of specific periods of gestational weight gain (GWG) with the category of VTE, pulmonary embolism (PE), or deep venous thrombosis (DVT) with or without PE.

**Methods:**

In a retrospective case–control study conducted in Shanghai First Maternity and Infant Hospital from January 1, 2017 to September 30, 2021, cases of VTE within pregnancy or the first 6 postnatal weeks were identified. Controls without VTE were randomly selected from women giving birth on the same day as the cases, with 10 controls matched to each case. Total GWG and rates of early, mid, and late GWG values were standardized into z-scores, stratified by pre-pregnant body mass index (BMI). The adjusted odds ratios (aORs) and 95% confidence intervals (CIs) were estimated through multivariate logistic regression models.

**Results:**

There were 196 cases (14.4 per 10,000) of VTE within pregnancy or the first 6 postnatal weeks were identified. Higher total weight gain was associated with increased risks of PE (aOR, 13.22; 95% CI, 2.03–85.99) and VTE (OR, 10.49; 95% CI, 1.82–60.45) among women with underweight. In addition, higher total weight gain was associated with increased risk of PE (aOR, 2.06; 95% CI, 1.14–3.72) among women with healthy weight. Similarly, rate of higher early weight gain was associated with significantly increased risk for PE (aOR, 2.15; 95% CI, 1.05–4.42) among women with healthy BMI. The lower rate of late weight gain was associated with increased risks of PE (aOR, 7.30; 95% CI, 1.14–46.55) and VTE (OR, 7.54; 95% CI, 1.20–47.57) among women with underweight. No significant associations between maternal rate of mid GWG and increased risk for any category of VTE, PE, or DVT with or without PE were present, regardless of maternal pre-pregnant BMI.

**Conclusion:**

The GWG associations with the category of VTE, PE, or DVT with or without PE differ at different periods of pregnancy. In order to effectively improve maternal and child outcomes, intensive weight management that continues through pregnancy may be indispensable.

## Introduction

Deep vein thrombosis (DVT) and pulmonary embolism (PE) are collectively referred to as venous thromboembolic disease (VTE). Although the rate of maternal mortality has declined over the past few decades, VTE remains an important cause of maternal deaths (1, 2). The greatest risk lies in the postpartum period; a woman’s risk of VTE is increased by 6 times, with reported incidence ranging from 0.5 to 2.0 per 1,000 deliveries (3, 4). Approximately 75%–80% of cases of pregnancy-associated VTE are caused by DVT, and 20%–25% of cases are caused by PE (5). However, few studies have examined trends in the incidence of pregnancy-related VTE in China.

Several studies have already identified risk factors for pregnancy-related VTE, including advanced maternal age, greater body mass index (BMI), cesarean delivery, preeclampsia, postpartum hemorrhage, and newborns with low birth weight (1, 6–11). They help care providers to target the use of thromboprophylaxis to women at risk to maximize its benefit (12). However, few studies have evaluated gestational weight gain (GWG) as a risk factor for VTE (13, 14). Although insufficient and excessive maternal weight gain has been linked to increased risks of VTE (13, 14), they have often not accounted for the effects of weight gain during certain periods of pregnancy. Furthermore, the associations of specific periods of GWG with the category of VTE, PE, or DVT with or without PE have not been reported. In addition, few studies have examined trends in the incidence of pregnancy-related VTE in China, and these studies have shown variable results (15–18).

For this retrospective case–control study of women, our objective was to explore the incidence of pregnancy-related VTE in China, and evaluate the association of maternal weight gain in different periods of pregnancy with the category of VTE, PE, or DVT with or without PE.

## Materials and Methods

### Study Design

We performed a retrospective case–control study, using data on all prenatal visit and discharges from Shanghai First Maternity and Infant Hospital for the period of January 1, 2017 to September 30, 2021 to evaluate the effect of maternal weight gain in different periods of pregnancy on VTE at any site. Shanghai First Maternity and Infant Hospital is an obstetrics and gynecology specialist hospital serving all women in Shanghai and even the whole country. There is no restriction on pregnant women who come to the hospital for prenatal care and giving birth. One-sixth of pregnant women in Shanghai give birth here every year. All pregnant women in our hospital at the first prenatal visit will write an informed consent on whether to agree that the clinical information related to this pregnancy will be used for research. The data including maternal demographical characteristics, reproductive history, as well as clinical information related to this pregnancy were collected. This study was approved by the Ethics Committee of Shanghai First Maternity and Infant Hospital (reference number: KS2057).

### Sample Size

We calculated the sample size in the case group (N) and control group (rN) from the following formula:


$$\text{N}=\left(1+1/\text{r}\right)\bar{p}\bar{q}{\left(U_{\alpha }+U_{\beta }\right)}^{2}{\left(P_{1}-P_{0}\right)}^{2}$$

According to the results of a previous study, the incidence rate (*P_0_*) of pregnancies with abnormal weight gain during pregnancy is 70% (19). The odds ratio (OR) of VTE for abnormal weight gain during pregnancy was 2 (13, 14); *P*_1_ = *P*_0_ *OR* / (1-*P*_0_ + *P*_0_ *OR*), $\bar{p}=\left(P_{1}+rP_{0}\right)/\left(1+r\right)$ , $\bar{q}=1-\bar{p}$ ; *U*_0.05_ = 1.96 (for two-sided 0.05 level test), and *U*_0.1_=1.28 (for two-sided 0.1 level test). Controls to cases with a 10:1 ratio, *r* = 10. Finally, N was calculated as 157, and rN was calculated as 1,570.

### Study Population

**Figure 1:**
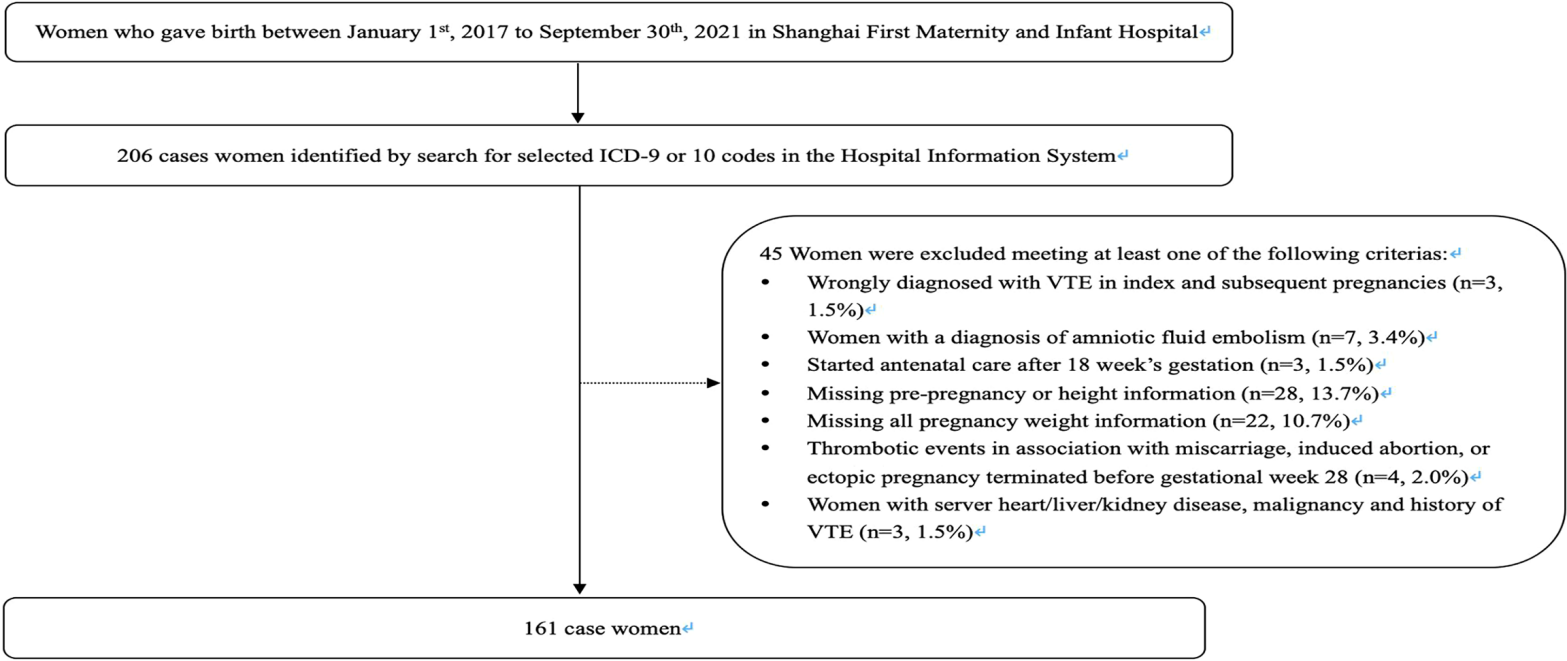
Study flow chart.

Cases were defined as women who had experienced an incident DVT or PE in pregnancy or within the 6 weeks following delivery. We identified 206 women registered with a diagnosis of VTE by searching for ICD-9 or ICD-10 codes in the Hospital Information System. We compared the search results identified from these codes with the number of VTE cases reported by the hospital within their historical quarterly reports to confirm the accuracy of these codes. We subsequently excluded 3 cases wrongly diagnosed with VTE in index and subsequent pregnancies and 7 possible cases with a diagnosis of amniotic fluid embolism. In addition, we excluded 4 cases with thrombotic events in association with miscarriage, induced abortion, or ectopic pregnancy terminated before gestational week 28. Further exclusions were applied to women who started antenatal care after 18 weeks’ gestation (*n* = 3); women who were missing data for pre-pregnancy, delivery weight, and height information (*n* = 28); and women who were missing all pregnancy weight information (*n* = 22). Finally, we excluded 3 cases with severe heart/liver/kidney disease, malignancy, or history of VTE. Some women fit into more than 1 exclusion category at the same time. The eligible case population comprised 161 women (**Figure 1**).

Controls without VTE in pregnancy or the first 6 weeks following delivery were randomly selected from women giving birth on the same day as the cases, with 10 controls matched to each case. Similarly, exclusions were applied to women who (1) induced abortion or ectopic pregnancy terminated before gestational week 28; (2) started antenatal care after 18 weeks’ gestation; (3) were missing data for pre-pregnancy, delivery weight, and height information; (4) were missing all pregnancy weight information; and (5) had severe heart/liver/kidney disease, malignancy, or history of VTE. A total of 1,610 controls were identified.

### Weight Measurements

Gestational age was estimated based on the date of last menstruation period and confirmed by first-trimester ultrasound date. Pre-pregnancy weight (kg) was based on self-reporting, while weight at every prenatal visit and at delivery was routinely measured to the nearest 0.1 kg using the available electronic weighting device, which should be calibrated every 6 months. Height (cm) at the first prenatal visit was routinely measured to the nearest 0.1 cm using the available electronic stadiometer in the hospital. Pre-pregnancy body mass index (BMI; kg/m^2^) was calculated as pre-pregnancy weight (kg) divided by height (m)^2^ and categorized as underweight (<18.5 kg/m^2^), healthy weight (18.5 to 23.9 kg/m^2^), overweight (24.0 to 27.9 kg/m^2^), and obese (≥28.0 kg/m^2^) according to Chinese BMI category standards (20, 21). However, due to the sporadic number of women with obesity, we analyze them together with women with overweight in this study.

We defined the following 3 gestational intervals: ≤14, 24 to 28, and >28 weeks. If a woman had more than 1 antenatal visit within an interval, we took her last weight measurements for that interval. Total GWG was calculated as last measurement of weight before delivery minus pre-pregnancy weight. However, if gestational age at last measurement of weight prior to delivery differs from gestational age at delivery by more than 7 days, the data were deleted. The rate of GWG in early pregnancy was calculated as early GWG (the antenatal weight up to ≤14 weeks minus the pre-pregnancy weight) divided by gestational weeks, the rate of GWG in mid pregnancy was calculated as mid GWG (weight measured within the 24 to 28 weeks’ gestational age interval minus the last weight measured ≤14 weeks) divided by the number of gestational weeks between the two weight measurements, and the rate of late pregnancy was calculated as late GWG (last measurement of weight prior to delivery minus weight measured within the 24 to 28 weeks’ gestational age interval) divided by the number of gestational weeks between the two weight measurements. Total GWG was standardized for gestational duration using BMI-specific GWG charts by gestational age for Chinese women (22). The means and standard deviations (SDs) corresponding to rates of GWG in early, mid, and late pregnancy for women in the control group were used to convert the rates of GWG values into z-scores, stratified by pre-pregnant BMI categories. All GWG and rates of GWG z-scores were first examined as continuous variables, and then categorized as <−1.0 (below), −1.0 to +1.0 (average), and >+1.0 (above) in data analyses.

### Exposure and Other Variables

Maternal demographics and lifestyle characteristics included maternal age (≥35 years or no), parity (0 or ≥1), education (college degree or above or no), assisted reproductive technology (ART) (yes or no), and pre-pregnancy BMI categories (underweight, normal, and overweight/obese).

Maternal pregnancy characteristics and complications included gestational age at delivery, delivery mode (vaginal delivery, planned cesarean section, and emergency cesarean section), multiple pregnancy (≥2), gestational diabetes mellitus (GDM), pregnancy induced hypertension (PIH), hypothyroidism, preterm birth (<37 week), postpartum hemorrhage (>500 ml after vaginal delivery, blood loss >1,000 ml after cesarean delivery), premature rupture of membranes, ischemic placental diseases [composed of preeclampsia, intrauterine growth retardation (IUGR), placental abruption, and stillbirth], placenta previa, abruptio placentae, and postpartum transfusion.

Newborn characteristics included fetal sex, birthweight, small for gestational age (SGA) ≤10th, large for gestational age (LGA) ≥90th according to Chinese sex- and gestational age-specific birth weight standards (23), macrosomia (>4,000 g), low birthweight (<2,500 g), very low birthweight (<1,500 g), sentinel congenital anomalies (atrial septal defect, ventricular septal defect, esophageal fistula, and hypospadias), hyperbilirubinemia (>12 mg/dl), and respiratory distress syndrome.

### Statistical Analyses

Maternal demographic characteristics and clinical factors were compared between venous thrombosis cases and control groups. Continuous variables were described by mean with standard deviation (SD) or median with interquartile range (IQR). Categorical variables were described by frequencies (%). Analysis of variance or Kruskal–Wallis H tests were performed for continuous data, and chi-square tests or Fisher’s exact tests were performed for categorical data.

Multivariate logistic regression models were used to estimate the adjusted odds ratios (aORs) and 95% confidence intervals (CIs) separately for PE alone, DVT with or without PE, and all VTE events across GWG in different periods of pregnancy. Regression models were adjusted for only co-variables with *p* < 0.2 (maternal age, parity, ART, delivery mode, fetal number, birthweight, PIH, GDM, hypothyroidism, length of gestation, postpartum hemorrhage, ischemic placental diseases, abruptio placentae, postpartum transfusion, infant gender, and sentinel congenital anomalies). *p*-value corresponding to the co-variable was calculated by the logistic regression model when estimating the aORs separately for PE alone, DVT with or without PE, and all VTE events across GWG in certain periods of pregnancy. **Tables 3**–**6** show different factors adjusted for women of different BMIs. Some studies showed that GWG has a correlation with maternal pre-pregnancy BMI, birthweight, maternal age, and length of gestation (24, 25). Therefore, interaction effects between GWG and these covariates were also tested to determine whether they needed to be brought into the regression model for adjustment.

All analyses were performed using the Statistical Analysis System (SAS) for Windows, version 9.4 (SAS Institute, Cary, NC). *p* < 0.05 was considered statistically significant.

### Patient and Public Involvement

Patients or the public were not involved in the design, or conduct, or reporting, or dissemination plans of our research.

## Results

### Study Population and Characteristics

A total of 135,696 women gave birth in Shanghai First Maternity and Infant Hospital between January 1, 2017 and September 30, 2021 and 196 cases (14.4 per 10,000) experienced VTE in pregnancy or in the first 6 postnatal weeks. Of these, PE alone accounted for 65.8% of VTE cases (106 of 161) and DVT alone or combined with PE accounted for 34.2% of cases (55 of 161). A total of 1,610 women who did not experience a VTE were selected as control. The prevalence of known risk factors for VTE was significantly higher in cases than controls (**Table 1**). In particular, cases were more likely to be of advanced age (22.4% versus 11.9%), to be overweight or obese (22.6% versus 16.5%), and to have delivery *via* emergency cesarean section (38.5% versus 6.4%), multiple birth (6.2% versus 1.6%), PIH (18.0% versus 5.2%), hypothyroidism (9.4% versus 4.5%), preterm birth (16.3% versus 5.2%), postpartum hemorrhage (13.7% versus 2.1%), ischemic placental diseases (26.1% versus 10.5%), abruptio placentae (3.7% versus 0.4%), postpartum transfusion (3.1% versus 0.6%), newborns with low birthweight (14.3% versus 3.7%), and very low birthweight (2.5% versus 0.3%). In addition, among cases, newborns of women had a higher prevalence of female gender (57.1% versus 47.1%), sentinel congenital anomalies (3.7% versus 1.1%), hyperbilirubinemia (14.9% versus 8.1%), and respiratory distress syndrome (6.2% versus 1.1%) than that of women in the control group.

**Table 1:** Characteristics of cases and controls.

Characteristic	Controls (*n* = 1610)	Cases (*n* = 161)	*p*-value
**Maternal age (years)**	30.0 ± 4.2	32.0 ± 4.2	<0.0001
**Maternal age ≥35 years, *n* (%)**	191 (11.9)	36 (22.4)	0.0002
**Nulliparous, *n* (%)**	1,290 (80.3)	119 (73.9)	0.0538
**Education, college degree and above, *n* (%)**	1,488 (92.4)	146 (90.7)	0.4648
**ART, *n* (%)**	81 (5.0)	4 (2.5)	0.1489
**Pre-pregnancy BMI (kg/m^2^), mean ± SD**	21.5 ± 3.0	21.9 ± 3.0	0.2475
**Pre-pregnancy BMI categories, *n* (%)**			
Underweight (<18.5 kg/m^2^)	209 (13.0)	10 (6.3)	0.0044
Healthy weight (18.5–23.9 kg/m^2^)	1,133 (70.5)	113 (71.1)	
Overweight and obese (≥24 kg/m^2^)	266 (16.5)	36 (22.6)	
**Gestational age at delivery (week), median (IQR)**	39 (38, 40)	38 (37, 39)	<0.0001
**Mode of delivery**			
Vaginal birth	906 (56.3)	29 (18.0)	<0.0001
Planned cesarean section	597 (37.2)	70 (43.5)	
Emergency cesarean section	102 (6.4)	62 (38.5)	
**Multiple pregnancy**	26 (1.6)	10 (6.2)	<0.0001
**Pregnancy complications**			
GDM	172 (10.7)	20 (12.4)	0.5022
PIH	83 (5.2)	29 (18.0)	<0.0001
Hypothyroidism	72 (4.5)	15 (9.4)	0.0068
Preterm birth	84 (5.2)	26 (16.3)	<0.0001
Postpartum hemorrhage	33 (2.1)	22 (13.7)	<0.0001
Ischemic placental diseases	169 (10.5)	42 (26.1)	<0.0001
Premature rupture of membranes	327 (20.3)	33 (20.5)	0.9614
Placenta previa	20 (1.2)	4 (2.5)	0.1946
Abruptio placentae	6 (0.4)	6 (3.7)	<0.0001
**Postpartum transfusion**	10 (0.6)	5 (3.1)	<0.0001
**Infant Gender (female)**	758 (47.1)	92 (57.1)	0.0151
**Birth Weight (g) (mean ± SD)**	3,325 ± 459	3,180 ± 650	0.0561
**SGA ≤10th**	96 (6.1)	10 (6.2)	0.9549
**LGA ≥90th**	151 (9.6)	17 (10.6)	0.6932
**Macrosomia (>4,000 g)**	91 (5.7)	8 (5.0)	0.7047
**Low birthweight (<2,500 g)**	59 (3.7)	23 (14.3)	<0.0001
**Very low birthweight (<1,500 g)**	5 (0.3)	4 (2.5)	0.0002
**Sentinel congenital anomalies**	17 (1.1)	6 (3.7)	0.0044
**Hyperbilirubinemia**	130 (8.1)	24 (14.9)	0.0034
**Respiratory distress syndrome**	18 (1.1)	10 (6.2)	<0.0001

ART, assisted reproductive technology; BMI, body mass index; GDM, gestational diabetes mellitus; PIH, pregnancy induced hypertension; SGA, small for gestational age; LGA, large for gestational age; IQR, median with interquartile range.

### Total Weight Gain During Pregnancy

In women with underweight, total GWG was 17.3 ± 6.9 kg in cases with PE, 22.1 ± 4.9 kg in cases with DVT either alone or combined with PE, 18.3 ± 6.6 kg in all cases of VTE, and 15.2 ± 5.5 kg in control (**Table 2**). Higher total weight gain was associated with increased risks of PE (aOR, 13.22; 95% CI, 2.03–85.99) and VTE (OR, 10.49; 95% CI, 1.82–60.45), while no evidence of an association between higher total weight gain and DVT was found among women with underweight (aOR, 6.21; 95% CI, 0.25–156.33). The associations of lower total GWG with PE, DVT, or VTE attenuated towards non-significant during pregnancy, regardless of pre-pregnancy BMI (**Table 3**). As for women with a BMI in the recommended range, total GWG was 15.3 ± 5.0 kg in cases with PE, 12.8 ± 4.5 kg in cases with DVT either alone or combined with PE, 14.5 ± 5.0 kg in all cases of VTE, and 14.7 ± 4.9 kg in control (**Table 1**), and did significantly differ between PE and control group (adjusted estimated mean difference combined 0 kg, 95% CI 0–0.01 kg, *p* = 0.0248) (**Table S1**). Higher total weight gain was associated with increased risk of PE (aOR, 2.06; 95% CI, 1.14–3.72), whereas there was no significant association between higher values and DVT (aOR, 0.43; 95% CI, 0.10–1.89) or VTE (aOR, 1.26; 95% CI, 0.69–2.29) among women with healthy weight. In addition, higher total GWG was not significantly associated with PE, DVT, or all VTE among women with overweight and obese (**Table 2**).

**Table 2:** Gestational weight gain at different periods of pregnancy.

	Control (*n* = 1,610)	PE (*n* = 106)	DVT with PE or without PE (*n* = 55)	All VTE (*n* = 161)
**Total GWG in pregnancy by pre-pregnant BMI categories**				
All women	14.4 ± 5.2	15.0 ± 5.3	12.4 ± 4.8	14.2 ± 5.3
Underweight (<18.5 kg/m^2^)	15.2 ± 5.5	17.3 ± 6.9	22.1 ± 4.9	18.3 ± 6.6
Healthy weight (18.5–23.9 kg/m^2^)	14.7 ± 4.9	15.3 ± 5.0	12.8 ± 4.5	14.5 ± 5.0
Overweight and obese (≥24 kg/m^2^)	12.8 ± 5.9	13.2 ± 5.5	10.5 ± 4.0	12.0 ± 5.0
**Rate of GWG in early pregnancy by pre-pregnant BMI categories**				
All women	0.14 ± 0.27	0.19 ± 0.27	0.12 ± 0.20	0.17 ± 0.25
Underweight (<18.5 kg/m^2^)	0.19 ± 0.36	0.43 ± 0.27	−0.07	0.33 ± 0.32
Healthy weight (18.5–23.9 kg/m^2^)	0.14 ± 0.24	0.17 ± 0.28	0.09 ± 0.16	0.15 ± 0.26
Overweight and obese (≥24 kg/m^2^)	0.09 ± 0.30	0.20 ± 0.22	0.20 ± 0.26	0.20 ± 0.23
**Rate of GWG in mid pregnancy by pre-pregnant BMI categories**				
All women	0.49 ± 0.16	0.52 ± 0.17	0.45 ± 0.17	0.50 ± 0.17
Underweight (<18.5 kg/m^2^)	0.49 ± 0.13	0.64 ± 0.06	0.85	0.69 ± 0.12
Healthy weight (18.5–23.9 kg/m^2^)	0.50 ± 0.16	0.53 ± 0.17	0.49 ± 0.14	0.52 ± 0.16
Overweight and obese (≥24 kg/m^2^)	0.45 ± 0.18	0.45 ± 0.17	0.33 ± 0.13	0.40 ± 0.16
**Rate of GWG in late pregnancy by pre-pregnant BMI categories**				
All women	0.54 ± 0.20	0.50 ± 0.22	0.42 ± 0.24	0.47 ± 0.23
Underweight (<18.5 kg/m^2^)	0.54 ± 0.21	0.42 ± 0.15	0.84 ± 0.07	0.52 ± 0.23
Healthy weight (18.5–23.9 kg/m^2^)	0.54 ± 0.19	0.53 ± 0.21	0.43 ± 0.23	0.50 ± 0.22
Overweight and obese (≥24 kg/m^2^)	0.52 ± 0.21	0.41 ± 0.24	0.34 ± 0.23	0.38 ± 0.24

GWG, gestational weight gain; BMI, body mass index; DVT, deep venous thrombosis; PE, pulmonary embolus; VTE, venous thromboembolism.

**Table 3:** Maternal total pregnancy weight gain by z-score categories with adjusted odds ratios for PE alone, DVT (including DVT and concomitant PE), and all VTE events.

Pre-pregnant BMI category	Total Weight Gain	Control (*n* = 1604)	PE (*n* = 101)	Adjusted odds ratio (95% CI)	DVT with PE or without PE (*n* = 50)	Adjusted odds ratio (95% CI)	All VTE (*n* = 151)	Adjusted odds ratio (95% CI)
	z-score category							
Underweight ^a^ (<18.5 kg/m^2^)	<−1	16	1	5.89 (0.50, 69.28)	0	NA	1	5.86 (0.52, 66.59)
	−1 to 1	175	3	Ref	1	Ref	4	Ref
	>1	18	3	13.22 (2.03, 85.99) ^*^	1	6.21 (0.25, 156.33)	4	10.49 (1.82, 60.45) ^*^
Healthy weight ^b^ (18.5–23.9) kg/m^2^	<−1	154	9	0.88 (0.40, 1.94)	9	1.93 (0.80, 4.62)	18	1.07 (0.57, 1.99)
	−1 to 1	828	49	Ref	21	Ref	70	Ref
	>1	148	17	2.06 (1.14, 3.72) ^*^	2	0.43 (0.10, 1.89)	19	1.26 (0.69, 2.29)
Overweight and obese ^c^ (≥24 kg/m^2^)	<−1	42	2	1.06 (0.21, 5.30)	2	0.78 (0.14, 4.18)	4	0.82 (0.24, 2.84)
	−1 to 1	187	14	Ref	13	Ref	27	Ref
	>1	36	3	0.81 (0.16, 4.16)	1	0.65 (0.08, 5.63)	4	0.81 (0.22, 3.04)

BMI, body mass index; DVT, deep venous thrombosis; PE, pulmonary embolus; VTE, venous thromboembolism.

a The analysis was adjusted for maternal age, fetal number, birthweight, postpartum hemorrhage, length of gestation, and abruptio placentae.

b The analysis was adjusted for maternal age, parity, ART, delivery mode, fetal number, birthweight, PIH, hypothyroidism, postpartum hemorrhage, length of gestation, ischemic placental diseases, abruptio placentae, infant gender, and sentinel congenital anomalies.

c The analysis was adjusted for maternal age, delivery mode, PIH, postpartum hemorrhage, length of gestation, ischemic placental diseases, and infant gender.

### Weight Gain During Early Pregnancy

In women with a BMI in the recommended range, rate of early GWG was 0.17 ± 0.28 kg/week in cases with PE, 0.09 ± 0.16 kg/week in cases with DVT either alone or combined with PE, 0.15 ± 0.26 kg/week in all cases of VTE, and 0.14 ± 0.24 kg/week in control (**Table 2**). Rate of higher early weight gain was associated with increased risk for PE (aOR, 2.15; 95% CI, 1.05–4.42), but not for DVT (aOR, 0.66; 95% CI, 0.15–3.00) and VTE (aOR, 1.42; 95% CI, 0.71–2.82) among women with healthy BMI (**Table 4**). The associations of lower rate of early GWG with PE, DVT, or VTE attenuated towards non-significant during pregnancy (**Table 4**). As for women with underweight or with overweight and obese, rate of early weight gain above average was not significantly associated with any category of VTE, PE, or DVT with or without PE (**Table 4**).

**Table 4:** Maternal early pregnancy weight gain by z-score categories with adjusted odds ratios for PE alone, DVT (including DVT and concomitant PE), and all VTE events.

Pre-pregnant BMI category	Rate of Early Weight Gain	Control (*n* = 1,537)	PE (*n* = 85)	Adjusted odds ratio (95% CI)	DVT with PE or without PE (*n* = 40)	Adjusted odds ratio (95% CI)	All VTE (*n* = 125)	Adjusted odds ratio (95% CI)
	z-score category							
Underweight ^a^ (<18.5 kg/m^2^)	<−1	5	0	NA	0	NA	0	NA
	−1 to 1	187	3	Ref	1	Ref	4	Ref
	>1	8	1	6.49 (0.56, 74.61)	0	NA	1	5.53 (0.47, 64.87)
Healthy weight ^b^ (18.5–23.9 kg/m^2^)	<−1	89	8	2.09 (0.92, 4.74)	3	1.37 (0.38, 4.96)	11	1.91 (0.90, 4.07)
	−1 to 1	891	47	Ref	22	Ref	69	Ref
	>1	107	11	2.15 (1.05, 4.42)^*^	2	0.66 (0.15, 3.00)	13	1.42 (0.71, 2.82)
Overweight and obese ^c^ (≥24 kg/m^2^)	<−1	30	0	NA	0	NA	0	NA
	−1 to 1	195	12	Ref	9	Ref	21	Ref
	>1	25	3	1.08 (0.24, 4.85)	3	3.01 (0.63, 14.41)	6	1.61 (0.51, 5.10)

BMI, body mass index; DVT, deep venous thrombosis; PE, pulmonary embolus; VTE, venous thromboembolism.

a The analysis was adjusted for fetal number, birthweight, and length of gestation.

b The analysis was adjusted for maternal age, ART, delivery mode, fetal number, birthweight, GDM, PIH, hypothyroidism, postpartum hemorrhage, length of gestation, ischemic placental diseases, abruptio placentae, infant gender, and sentinel congenital anomalies.

c The analysis was adjusted for maternal age, delivery mode, PIH, postpartum hemorrhage, length of gestation, ischemic placental diseases, and infant gender.

### Weight Gain During Mid and Late Pregnancy

No significant associations between maternal rate of mid GWG and increased risk for any category of VTE, PE, or DVT with or without PE were present, regardless of maternal pre-pregnant BMI (**Table 5**).

**Table 5:** Maternal mid pregnancy weight gain by z-score categories with adjusted odds ratios for PE alone, DVT (including DVT and concomitant PE), and all VTE events.

Pre-pregnant BMI category	Rate of Mid Weight Gain	Control (*n* = 1,538)	PE (*n* = 80)	Adjusted odds ratio (95% CI)	DVT with PE or without PE (*n* = 35)	Adjusted odds ratio (95% CI)	All VTE (*n* = 115)	Adjusted odds ratio (95% CI)
	z-score category							
Underweight ^a^ (<18.5 kg/m^2^)	<−1	40	0	NA	0	NA	0	NA
	−1 to 1	130	2	Ref	0	Ref	2	Ref
	>1	31	1	1.08 (0.07, 17.49)	1	NA	2	2.78 (0.30, 26.09)
Healthy weight ^b^ (18–5-23.9) kg/m^2^	<−1	155	9	1.08 (0.49, 2.40)	6	1.82 (0.66, 5.00)	15	1.26 (0.66, 2.41)
	−1 to 1	782	40	Ref	16	Ref	56	Ref
	>1	151	14	1.28 (0.61, 2.67)	1	0.25 (0.03, 1.93)	15	0.96 (0.47, 1.92)
Overweight and obese ^c^ (≥24 kg/m^2^)	<−1	34	1	0.49 (0.05, 5.01)	3	1.75 (0.39, 7.93)	4	1.12 (0.32, 3.92)
	−1 to 1	176	12	Ref	8	Ref	20	Ref
	>1	39	1	0.33 (0.03, 3.35)	0	NA	1	0.21 (0.02, 1.86)

BMI, body mass index; DVT, deep venous thrombosis; PE, pulmonary embolus; VTE, venous thromboembolism.

a The analysis was adjusted for fetal number, birthweight, PIH, and ischemic placental diseases.

b The analysis was adjusted for maternal age, parity, ART, delivery mode, fetal number, birthweight, PIH, hypothyroidism, postpartum hemorrhage, length of gestation, ischemic placental diseases, abruptio placentae, infant gender, and sentinel congenital anomalies.

c The analysis was adjusted for maternal age, delivery mode, PIH, postpartum hemorrhage, length of gestation, ischemic placental diseases, and infant gender.

In women with underweight, the rate of late GWG was 0.42 ± 0.15 kg/week in cases with PE, 0.84 ± 0.07 kg/week in cases with DVT either alone or combined with PE, 0.52 ± 0.23 kg/week in all cases of VTE, and 0.54 ± 0.21 kg/week in control (**Table 2**). The lower rate of late weight gain was associated with increased risks of PE (aOR, 7.30; 95% CI, 1.14–46.55) and VTE (OR, 7.54; 95% CI, 1.20–47.57) among women with underweight. The association between the higher rate of late weight gain and PE, DVT, and VTE was not significant, regardless of maternal pre-pregnant BMI (**Table 6**).

**Table 6:** Maternal late pregnancy weight gain by z-score categories with adjusted odds ratios for PE alone, DVT (including DVT and concomitant PE), and all VTE events.

Pre-pregnant BMI category	Rate of Late Weight Gain	Control (*n* = 1,602)	PE (*n* = 94)	Adjusted odds ratio (95% CI)	DVT with PE or without PE (*n* = 48)	Adjusted odds ratio (95% CI)	All VTE (*n* = 142)	Adjusted odds ratio (95% CI)
	z-score category							
Underweight ^a^ (<18.5 kg/m^2^)	<−1	18	2	7.30 (1.14, 46.55)^*^	0	NA	2	7.54 (1.20, 47.57)^*^
	−1 to 1	201	4	Ref	0	Ref	4	Ref
	>1	29	0	NA	2	NA	2	2.20 (0.24, 19.90)
Healthy weight ^b^ (18.5–23.9 kg/m^2^)	<−1	156	10	1.03 (0.49, 2.20)	9	1.97 (0.81, 4.74)	19	1.25 (0.69, 2.25)
	−1 to 1	799	49	Ref	20	Ref	69	Ref
	>1	145	10	0.95 (0.41, 2.18)	2	0.52 (0.11, 2.37)	12	0.83 (0.39, 1.75)
Overweight and obese ^c^ (≥24 kg/m^2^)	<−1	45	5	1.62 (0.47, 5.54)	6	2.09 (0.60, 7.29)	11	1.98 (0.78, 5.01)
	−1 to 1	170	12	Ref	8	Ref	20	Ref
	>1	40	1	0.22 (0.03, 1.87)	1	0.31 (0.03, 2.85)	2	0.28 (0.06, 1.35)

BMI, body mass index; DVT, deep venous thrombosis; PE, pulmonary embolus; VTE, venous thromboembolism.

a The analysis was adjusted for fetal number, birthweight, PIH, and ischemic placental diseases.

b The analysis was adjusted for maternal age, delivery mode, fetal number, birthweight, PIH, hypothyroidism, postpartum hemorrhage, length of gestation, ischemic placental diseases, abruptio placentae, infant gender, and sentinel congenital anomalies.

c The analysis was adjusted for maternal age, delivery mode, PIH, postpartum hemorrhage, ischemic placental diseases, and infant gender.

## Discussion

### Main Findings

In this study, we found different associations of gestational stage-specific weight gain with venous thrombosis. Of those, higher total weight gain was associated with increased risks of PE and VTE among women with underweight. In addition, the rates of higher early and total weight gain were associated with increased risk of PE among women with healthy BMI. The lower rate of late weight gain was associated with increased risks of PE and VTE as for women with underweight only.

### Strengths and Limitations

There are strengths in our study. Due to the detailed clinical data, such as pre-pregnancy weight, weight measurements at every prenatal visit, and weight measurements before delivery beyond the registry, it was possible for us to study both weight gain in different periods of pregnancy and to take the differences in types of VTE (e.g., VTE, PE, or DVT with or without PE) into account. Moreover, the use of total weight gain z-scores, which was standardized for gestational duration using BMI-specific GWG charts, or the use of the rate of GWG z-scores instead of weight gain in kilograms could help to disentangle the effects of pregnancy weight gain on VTE from the effects of gestational duration, because GWG is highly correlated to the gestational duration. Our study extends previous studies by accounting for effect modification by pre-pregnancy BMI and using a gestational age-independent measure of pregnancy weight gain. In addition, GWG z-scores were stratified by pre-pregnant BMI based on Chinese BMI category standards (22), instead of World Health Organization (WHO) BMI categories, which are not available specifically for Asians, who have a higher body fat ratio and risk of obesity-related diseases than Europeans with the same BMI.

There are also limitations in our study. The number of VTE was decreased when stratified by pre-pregnant BMI categories, especially among women with obesity. For this reason, we analyzed the effect of weight gain during pregnancy on VTE in women with obesity together with women with overweight. Furthermore, the incidence of VTE might be underestimated in this study, since women who had high risks of VTE during pregnancy shown in **Table S2** typically receive low-molecular-weight heparin (LMWH), thus reducing the incidence of developing DVT in the postpartum period for these women. The limitation of not all women having a weight recorded <14 weeks or 24 to 28 weeks’ gestational age is not addressed, meaning that the rate of weight gain in early, mid, and late pregnancy could not be calculated for all women.

### Interpretation

GWG has been suggested to be associated with subsequent risks of adverse pregnancy outcomes, such as preterm birth, pre-eclampsia, and caesarean section (24, 26), but evidence to clarify the relationship between GWG and maternal VTE has been sporadic (13, 14). A Norwegian hospital case–control study reported that large weight gains (>90th percentile or >21.0 kg) were associated with 60% increased odds of postpartum VTE, while small maternal weight gain is an independent antenatal risk factor for VTE (13). In a Washington State (USA) population-based case–control study, women with large weight gains during pregnancy (>22 kg), independently of BMI, were more likely to have VTE (14). In line with these studies, we observed that higher total GWG was associated with higher risk of PE and VTE among women with underweight. In addition, the rate of higher total weight gain was associated with higher risk of PE in women with a BMI in the recommended range. Significantly, the regression model was adjusted for only co-variables with *p* < 0.2. Although there was no significant difference in the prevalence of parity, ART, and GDM between the case and control group (**Table 1**), we still included these factors and tested whether they need to be included in the regression model for adjustment, because some studies reported that nulliparous women, women subjected to ART, and women with GDM were at increased risk for pregnancy-related VTE (13, 27). In contrast, hyperbilirubinemia and respiratory distress syndrome have been not adjusted for, because they appear after delivery. Overweight gain during pregnancy accompanied with increased intraabdominal pressure can encourage blood stasis through iliac vessel compression. Furthermore, elevated inflammatory cytokines and adipokines with increased fat deposition promote endothelial dysfunction and platelet hyperreactivity. Fat deposition also skews the hemostatic–fibrinolytic balance through elevation of procoagulant factors including von Willebrand factor, fibrinogen, factor VII, factor VIII, issue factor, and impairment of fibrinolysis by elevation of plasminogen activator inhibitor (28). On top of differential inflammatory responses, women with high weight have longer durations of labor, and greater rates of chorioamnionitis, postpartum hemorrhage, and surgical complications, which may all lead to the observed greater risk of VTE after delivery (29).

There is growing recognition that the impacts of gestational stage-specific weight gain on pregnancy outcomes may vary (30–34). Ravi et al. evaluated the associations of weight gain within each of the 10 gestational intervals with infant birth weight and found that maternal weight only in the first half of gestation is a determinant of infant birth weight (34). Similarly, Gaillard et al. examined the associations of maternal weight gain in early, mid, and late pregnancy with childhood cardio-metabolic risk factors separately and established that higher weight gain in early pregnancy is associated with an adverse cardio-metabolic profile in offspring (30). GWG in early pregnancy largely reflects maternal fat deposition, whereas GWG in mid and late pregnancy is also attributed to maternal and amniotic fluid expansion, and growth of the fetus, placenta, and uterus (35). In this study, we found that higher GWG in early pregnancy was associated with higher risks of PE in women with healthy weight. Studies have found that mothers with increased fat deposition during early pregnancy may involve the multifactorial engagement of alterations to blood flow, hypercoagulability, chronic low-grade inflammation, and endothelial dysfunction, which may lead to PE (28, 36). Therefore, GWG in early pregnancy, prior to the development of pregnancy outcomes, might be as important as, or more important than, GWG in late pregnancy with respect to pregnancy outcomes.

The rate of late GWG below average in mother with underweight was also associated with an increased risk of PE and VTE in our analysis. This finding, if true, could result from low amniotic fluid volume and fetal weight by ischemic placental disease (including preeclampsia, intrauterine growth retardation, stillbirth, and placental abruption), perhaps a potential for embolism, leading to PE (1). Blondon et al. found that the delivery of a newborn with low birth weight is associated with a 3-fold increased risk of maternal postpartum VTE (1). In another Norwegian hospital-based case–control study, mothers of newborns with IUGR were at 3.8-fold risk of postpartum VTE (13). Moreover, hypertension during pregnancy and preeclampsia are also associated with an increased VTE risk during pregnancy and postpartum period (9, 37). However, a randomized clinical trial indicated that pre-pregnancy weight loss intervention has favorable effects on the early intrauterine environment (38). Lifestyle intervention during pregnancy could, to some extent, limit GWG and improve maternal and infant health (39). Therefore, pre-pregnancy weight interventions integrated into intensive weight management that continues through pregnancy may be indispensable to decrease the risks of PE.

There was no statistical association between maternal weight gain and DVT across all pre-pregnant BMI categories. Similarly, Matthew et al. retrospectively analyzed a large database from America and found that only the risk of PE is elevated in patient classification as heavier categories after surgery, whereas there was no positive association between DVT and BMI (40, 41). Explanations for this observed association exist, of which anticoagulation used for VTE prophylaxis during pregnancy is most plausible. All pregnant women in Shanghai are managed based on Royal College of Obstetricians and Gynaecologists (RCOG) Green-top Guidelines (42) and Queensland Clinical Guidelines (43, 44). Briefly, they will undergo a documented assessment of risk factors for VTE throughout pregnancy, intrapartum, and the puerperium. Any woman with risk factors shown in **Table S2** should be considered for prophylactic LMWH. Previous studies have evaluated the efficacy of LMWH for thromboprophylaxis, revealing that LMWH probably results in little to no difference in the incidence of PE in patients undergoing knee arthroscopy, but reduces the risk of asymptomatic DVT (45). Similarly, eight RCTs showed no clear differences between the LMWH and no prophylaxis or placebo groups in patients with lower-limb immobilization for PE, but less DVT in the LMWH groups (46). Therefore, aggressive pharmacologic anticoagulation regimens during pregnancy can decrease the DVT rate but have not been shown to affect the rate of PE. Meanwhile, common risk factors for DVT, like history of multiple deliveries, smoking, and obesity, are less frequently observed in China (16, 47). These may be the reasons for the higher incidence of PE in this study. Approximately 75%–80% of cases of pregnancy-associated VTE are caused by DVT, and 20%–25% of cases are caused by PE (5), whereas over 65% of cases in this study were caused by PE (106 out of 161 cases). However, the evidence is very uncertain, and further high-quality very-large-scale randomized trials are needed to determine the effects of currently used treatments in women with different VTE risk factors.

## Conclusion

The GWG associations with the category of VTE, PE, or DVT with or without PE differ at different periods of pregnancy. In order to effectively improve maternal and child outcomes, pre-pregnancy weight interventions integrated into intensive weight management that continues through pregnancy may be indispensable.

## Data Availability Statement

The raw data supporting the conclusions of this article will be made available by the authors, without undue reservation.

## Ethics Statement

This study was approved by Ethics Committee of Shanghai First Maternity and Infant Hospital (reference number: KS2057). The patients/participants provided their written informed consent to participate in this study.

## Author Contributions

YW, JP, and LD participated in interpretation of data and involved in drafting the manuscript. ZZ, TZ, XZ, ZH, and RC analyzed the data and critically revised the manuscript. XH made substantial contributions to conception and design, interpreted the data, and critically revised the manuscript. All authors read and approved the final manuscript.

## Funding

This research was supported by the National Natural Science Foundation of China (81873816 and 82071629), the Foundation of Shanghai Municipal Health Commission (202040128), the Pudong Commission of Health and Family Planning (PW2019D-13), and Clinical research plan of SHDC (SHDC2020CR6021).

## Conflict of Interest

The authors declare that the research was conducted in the absence of any commercial or financial relationships that could be construed as a potential conflict of interest.

## Publisher’s Note

All claims expressed in this article are solely those of the authors and do not necessarily represent those of their affiliated organizations, or those of the publisher, the editors and the reviewers. Any product that may be evaluated in this article, or claim that may be made by its manufacturer, is not guaranteed or endorsed by the publisher.
