# Supplementary material for: Association Between Maternal Weight Gain in Different Periods of Pregnancy and the Risk of Venous Thromboembolism: A Retrospective Case–Control Study
Source: Front Endocrinol (Lausanne). 2022 Jul 18;13:858868. doi: 10.3389/fendo.2022.858868 (PMC9339610; doi:10.3389/fendo.2022.858868)
Supplement: Supplementary file 1 [file Table_1.docx]

Table S1 Absolute effect size of gestational weight gain at different periods of pregnancy.

|  | **PE**  **Adjusted effect size (95% CI)**  **(n=99)** | ***P* value** | **DVT with PE or without PE**  **Adjusted effect size (95% CI)**  **(n=52)** | ***P* value** | **All VTE**  **Adjusted effect size (95% CI)**  **(n=151)** | ***P* value** |
| --- | --- | --- | --- | --- | --- | --- |
| **Total GWG in pregnancy by pre-pregnant BMI categories** | | | | | | |
| Underweight (< 18.5 kg/m2) | 0.01 (0, 0.04) | 0.2415 | 0 (0, 0.03) | 0.5758 | 0.01 (0, 0.05) | 0.1400 |
| Normal weight (18.5-23.9 kg/m2) | **0 (0, 0.01) ^*^** | **0.0248** | **0 (0, 0.01)^*^** | **0.0285** | 0 (0, 0.01) | 0.5423 |
| Overweight and obese (≥ 24 kg/m2) | 0 (0, 0.02) | 0.5960 | 0 (0, 0.01) | 0.9452 | 0. (0, 0.02) | 0.7117 |
| **Rate of GWG in early pregnancy by pre-pregnant BMI categories** | | | | | | |
| Underweight (< 18.5 kg/m2) | 0.01 (0, 0.04) | 0.3047 | 0 (0, 0.04) | 0.4826 | 0 (0, 0.03) | 0.5079 |
| Normal weight (18.5-23.9 kg/m2) | 0 (0, 0.01) | 0.3686 | 0 (0, 0.01) | 0.2411 | 0 (0, 0.01) | 0.8911 |
| Overweight and obese (≥ 24 kg/m2) | 0 (0, 0.03) | 0.3744 | 0.01 (0, 0.04) | 0.2524 | 0.01 (0, 0.04) | 0.1726 |
| **Rate of GWG in mid pregnancy by pre-pregnant BMI categories** | | | | | | |
| Underweight (< 18.5 kg/m2) | 0.01 (0, 0.05) | 0.1372 | **0.02 (0, 0.07)^*^** | **0.0426** | **0.02 (0, 0.08)^*^** | **0.0229** |
| Normal weight (18.5-23.9 kg/m2) | 0 (0, 0.01) | 0.3989 | 0 (0, 0.01) | 0.8465 | 0 (0, 0.01) | 0.5895 |
| Overweight and obese (≥ 24 kg/m2) | 0 (0, 0.02) | 0.6243 | **0.01 (0, 0.06)^*^** | **0.0378** | 0.01 (0, 0.04) | 0.0922 |
| **Rate of GWG in late pregnancy by pre-pregnant BMI categories** | | | | | | |
| Underweight (< 18.5 kg/m2) | 0.01 (0, 0.04) | 0.2203 | 0 (0, 0.01) | 0.8407 | 0 (0, 0.04) | 0.2683 |
| Normal weight (18.5-23.9 kg/m2) | 0 (0, 0.01) | 0.5014 | **0.01 (0, 0.02)^*^** | **0.0028** | **0 (0, 0.01)^*^** | **0.0367** |
| Overweight and obese (≥ 24 kg/m2) | **0.02 (0, 0.07) ^*^** | **0.0075** | **0.05 (0.01, 0.10)^*^** | **0.0002** | **0.06 (0.02, 0.12)^*^** | **<0.0001** |

Abbreviations: GWG: gestational weight gain; BMI: body mass index; DVT: deep venous thrombosis; PE: pulmonary embolus; VTE: venous thromboembolism.

The co-variables adjusted by effect size were the same as that of regression model.

Table S2 Risk assessment for venous thromboembolism (VTE)

| **Risk factors for VTE** | **Score** |
| --- | --- |
| **Pre-existing risk factors** | |
| Previous VTE (except a single event related to major surgery) | 4 |
| Single previous VTE related to major surgery | 3 |
| High-risk thrombophilia: antithrombin deficiency; double heterozygous for prothrombin G20210A mutation and factor V Leiden; factor V Leiden homozygous or prothrombin G20210A mutation homozygous | 3 |
| Medical comorbidities e.g. cancer, heart failure, active SLE, IBD or inflammatory polyarthropathy, nephrotic syndrome, type I DM with nephropathy, sickle cell disease, current IVDU | 3 |
| Low-risk thrombophilia: factor V Leiden heterozygous; prothrombin G20210A heterozygous; protein C or protein S deficiency | 1 |
| Family history of unprovoked or estrogen-provoked VTE in first-degree relative | 1 |
| Age ≥35 | 1 |
| Pre-pregnant BMI 28-34.9kg/m^2^ | 1 |
| Pre-pregnant BMI ≥35 kg/m^2^ | 2 |
| Parity ≥3 | 1 |
| Smoker | 1 |
| Varicose veins of lower limb | 1 |
| IVF/ART | 1 |
| Multiple pregnancy | 1 |
| prediabetes or GDM (using insulin) | 1 |
| Current pre-eclampsia/eclampsia | 1 |
| Placental abruption | 1 |
| **Obstetric risk factors** | |
| Elective caesarean section | 1 |
| Caesarean section in labor | 2 |
| Hysterectomy | 2 |
| Preterm delivery in this pregnancy (< 37^+0^ weeks) | 1 |
| PPH > 1000 ml or blood transfusion | 1 |
| Stillbirth in this pregnancy | 1 |
| Operative delivery, e.g. forceps delivery, hydrostatic intrauterine balloon tamponade | 1 |
| Prolonged labour (> 24 hours) | 1 |
| **Transient risk factors** | |
| OHSS | 4 |
| Hyperemesis | 3 |
| Any surgical procedure in pregnancy or puerperium except immediate repair of the 3 perineum, e.g. appendicectomy, postpartum sterilization | 3 |
| Immobility ≥7 days | 2 |
| Immobility ≥2 days | 1 |
| Current systemic infection | 1 |
| Dehydration | 1 |

Abbreviations: ART assisted reproductive technology; IVF in vitro fertilization; OHSS ovarian hyperstimulation syndrome; VTE venous thromboembolism; PPH postpartum hemorrhage; BMI based on booking weight; LMWH low-molecular-weight heparin; DM = diabetes mellitus; IVDU intravenous drug user; IBD inflammatory bowel disease; GDM gestational diabetes melitus;

• If single score ≥ 3 antenatally, consider thromboprophylaxis from the first trimester or pre-pregnancy to 24 hours before delivery.

• If single score＜3, total score ≥ 4 antenatally, consider thromboprophylaxis from the first trimester to 24 hours before delivery.

• If single score＜3, total score ≥ 3 antenatally, consider thromboprophylaxis from 28 weeks to 24 hours before delivery.

• If total score ≥ 2 postnatally, consider thromboprophylaxis for at least 4 days

• If total score ≥ 3 postnatally, consider thromboprophylaxis for at least 7-10 days.
